# Cold Exposures in Relation to Dysmenorrhea among Asian and White Women

**DOI:** 10.3390/ijerph21010056

**Published:** 2023-12-30

**Authors:** Tianying Wu, Cassie Doyle, Joy Ito, Neeraja Ramesh, Deepali Karina Ernest, Noe C. Crespo, Fang-Chi Hsu, Eyal Oren

**Affiliations:** 1 Division of Epidemiology and Biostatistics, School of Public Health, San Diego State University, San Diego, CA 92182, USA; cassiemariedoyle@gmail.com (C.D.); jito@sdsu.edu (J.I.); nramesh5943@sdsu.edu (N.R.); eoren@sdsu.edu (E.O.); 2 Moores Cancer Center, School of Medicine, University of California, San Diego, CA 92037, USA; 3 Department of Epidemiology, Human Genetics & Environmental Sciences, School of Public Health, University of Texas Health Science Center at Houston, Houston, TX 77030, USA; dkernest98@gmail.com; 4 Division of Health Promotion and Behavioral Science, School of Public Health, San Diego State University, San Diego, CA 92182, USA; ncrespo@sdsu.edu; 5 Department of Biostatistics and Data Science, Division of Public Health Sciences, Wake Forest University School of Medicine, Winston-Salem, NC 27101, USA; fhsu@wakehealth.edu

**Keywords:** cold exposure, dysmenorrhea, Asian culture, women’s health

## Abstract

Dysmenorrhea is highly prevalent, ranging from 16% to 91% among women, and it can lead to multiple reproductive disorders. However, risk factors associated with dysmenorrhea remain unexamined. Cold exposures can significantly disturb blood circulation and prostaglandin production in the uterus, leading to dysmenorrhea. This study investigated the relationship between cold exposures and dysmenorrhea, as well as potential disparities between Asians and Whites and the potential cultural influences on these associations. This was a cross-sectional survey among 197 Asian and 222 non-Asian women recruited from the U.S., with more than 40% from California. We assessed cold exposures, such as the frequency of consumption of cold water/drinks and ice cream, as well as room temperatures at home and public places, for both summer and winter over the past 12 months. The type of cold exposure associated with dysmenorrhea differs between Asian and White women. We found that among Asian women, a higher frequency of ice cream consumption in winter (beta = 1.19, *p* = 0.0002 when comparing high to low categories) was associated with dysmenorrhea; however, among White women, increased consumption of cold water/drinks in winter (beta = 0.49, *p* = 0.04 when comparing high to low categories) was also associated with dysmenorrhea. Higher home room temperatures in winter were associated with reduced severity of dysmenorrhea among White women but not among Asian women. All these associations supported our hypothesis and were stronger among women who lived in states with colder winters. However, there are a few exceptions. For instance, women who drank cold water/drinks less frequently during their menstrual period were more likely to experience more severe dysmenorrhea. In conclusion, this study provides crucial evidence to support the link between cold exposures and dysmenorrhea among Asians and Whites. The associations contradictory to our hypothesis are likely due to reserved causation influenced by Asian cultural practice. This paper sheds light on an understudied area that profoundly affects women’s quality of life.

## 1. Introduction

Dysmenorrhea symptoms (menstrual pain) occurs in 16–91% women [1] and profoundly affects women’s quality of life, yet it remains considerably understudied. Dysmenorrhea has significant associations with endometriosis, infertility, and vascular diseases [2,3] and can often manifest without underlying pelvic diseases [4], making its study a valuable approach to preventing numerous gynecological and reproductive disorders in women.

While the exact causes of dysmenorrhea are not fully understood, cold exposures have been found to be associated with dysmenorrhea in women working in slaughter houses [5]. In healthy people, skin cooling starts to trigger pain and protective reactions at around 20 °C [6]. This is because the survival of both humans and animals depends on their ability to sense cooling, leading them to develop various strategies to mitigate and avoid low temperatures [6]. Several biological mechanisms, though complicated, have been proposed. These include but are not limited to, ion channel expression [6,7], nitric oxide availability [8], the production of prostaglandins [9,10], reduced perfusion of microcirculation [11], and disturbed blood circulation in the uterus [12,13] under cold temperatures. All of these factors can further accelerate sensation of pain [6,14,15]. However, cold exposures and women’s behavior during cold exposures in relation to dysmenorrhea among free living populations has not been well examined. Studying cold exposures among the free-living population is crucial as it will have wider implications. Examples of cold exposures include temperatures of foods and drinks, daily dress habits, and indoor room temperatures during cold seasons.

Traditional Asian cultures have long emphasized the importance of keeping warm during menstruation. For instance, in China, Korea, and Japan, women are advised to stay warm (e.g., by dressing warmly during cold weather) and avoid cold foods/drinks during their menstrual periods [16]. These cultural practices are strongly influenced by Traditional Chinese Medicine (TCM), which is more accepted and practiced in these countries. A similar cultural practice is well accepted in India [17], where Ayurvedic medicine shares some overlapping philosophies with TCM. Some previous randomized intervention studies indirectly support this practice, as they have demonstrated that heat therapy during the menstrual period can reduce dysmenorrhea [18,19]. In TCM, self-care is recognized as an essential component of both treatment and prevention [20,21]. TCM particularly emphasizes the thermal nature of food and drink choices in relation to women’s health. Women should avoid cold drinks and foods, especially for those who may be more susceptible to cold-related health issues. For example, TCM suggests that women who often experience cold hands and feet might be more prone to dysmenorrhea caused by cold water. The efficacy of this practice is well-documented in Armour et al. [21]. Regrettably, these traditional practices may be undermined by the process of westernization and acculturation following immigration to the U.S. Therefore, studying cold exposures with dysmenorrhea among Asian migrants and Asian non-migrants can help us determine whether these cold exposures may play an important role in dysmenorrhea among Asians and the impact of acculturation on cultural practice related to cold exposure between Asian migrants and Asian non-migrants.

Moreover, even though the relationship between cold exposures and dysmenorrhea might not be emphasized in all cultures, it is crucial to study this connection among different racial groups. Doing so will help determine whether sensitivity to cold exposures is unique to Asians or is universal across different races.

We aim to provide more evidence for evaluating certain traditional Asian philosophies, particularly those regarding TCM and its perspectives on eating and living habits in relation to cold exposure. This includes examining whether exposure to cold environments is associated with increased severity of dysmenorrhea. This assessed the relationships between general cold exposures and dysmenorrhea and if the types of cold exposures that are associated with dysmenorrhea differ among Asians and non-Asians.

## 2. Materials and Methods

### 2.1. Study Design, Population, and Recruitment

The Healthy Aging Survey (HAS) was launched in February 2022 with the aim of determining disparities in risk factors associated with aging between Asians and non-Asians. The data used for this paper were collected from 23 February 2022 to 20 May 2023. We included all racial groups aged 18 to 65. To mitigate the influence of specific infectious diseases, we excluded individuals with HIV, hepatitis, and active tuberculosis. To improve validity, participants were also excluded based on the following major criteria: completing less than 90% of the survey questions, finishing the survey in less than 15 min, inconsistencies between the birth gender reported in the screening survey and the formal survey, discrepancies between the birth date and age as of the enrollment date, a height of less than 60 cm, a weight of less than 50 pounds, and possession of a suspicious IP address. By May 20, a total of 885 participants had completed the survey. After applying our exclusion criteria, we ended with 617 participants, comprising 437 women and 180 men. Since this study centers on women who responded to reproductive health questions, including those about dysmenorrhea, only the data from the 419 female participants are included in this paper.

We enhanced our recruitment efforts among Asians, both locally and nationally, by implementing targeted strategies and expanding our outreach. For Asians in San Diego and southern California, we sought participants from various Asian-dense communities, including 4S Ranch, Camel Valley, Mira Mesa, and Rancho Penasquitos, through the distribution of flyers, participation in community outreach events, and leveraging our friend network. Furthermore, we engaged with specific segments within the Asian community or broader Asian organizations. For the Chinese community, we reached out to key platforms such as Chinese WeChat groups and the Chinese Bible Church in San Diego. To involve the Indian community, we collaborated with the San Diego Malayali Association, the Sri Lakshmi Venkateshwara Temple, and Indian grocery stores like Miramar Cash and Carry. We also established connections with Asian organizations, including Asian Americans and Pacific Islanders (APIDA) and San Diego Asian Americans for Equality (SDAAFE). In addition, we collaborated with student Asian organizations at San Diego State University (SDSU), such as (AAPI)phany of SDSU, Andrés Bonifacio Samahan, the Filipinx American Cultural Organization, and the SDSU Taiwanese Student Association.

Our nationwide recruitment efforts included recruitment through SDSU alumni networks, partnerships with ResearchMatch, Alliance for Impact (AFI), a non-profit national organization serving Asian communities, and the registry of the Collaborative Approach for Asian Americans, Native Hawaiians, and Pacific Islanders Research and Education (CARE). These comprehensive approaches allowed us to effectively engage with and recruit participants from diverse Asian backgrounds.

A typical U.S. survey [22] or cohort [23] has less than 5% Asians, which is why Asians are understudied. We oversampled Asians, resulting in 46% Asians, 34% Whites, and 20% other ethnic groups. Asian women in our survey are primarily composed of 33% Chinese, 21% Asian Indians, 15% Filipinos, 9% Vietnamese, and the remaining 22% from other Asian countries (e.g., Thai, Malaysian, Pakistani, Sri Lankan), excluding Southwest Asia countries. Among Asians, approximately 36% were Asian migrants, who were first-generation immigrants born outside of the U.S. and arrived in the U.S. after the age of 12. This survey enrolled both men and women, but we only present data among women.

### 2.2. Assessment of Types of Cold Exposures

This is a cross-sectional survey. We assessed different exposures in one survey, which included asking participants about their exposures during both summer and winter in the past year. We assessed both endogenous and exogenous cold exposures. Endogenous cold exposures mainly include temperatures of water/drinks and foods. Exogenous cold exposures encompass outdoor and indoor temperatures. Exact outdoor temperatures cannot be accurately evaluated without detailed climate data which was not the focus of this study. However, on average, temperatures are colder in winter than in summer. Thus, we asked about cold exposures in summer and winter. We inquired about indoor temperatures at home and in public places. In addition, choice of clothing can significantly influence the impact of room temperatures. We inquired about usual dress habits, and habits of wearing thermal pants (pants worn underneath regular pants) during cold months, which is particularly important for Asian culture.

#### 2.2.1. Assessment of Temperatures of Water/Drinks during Winter and Summer

We asked the frequency of participants drinking cold and hot water or drinks (milk, juice, tea, etc.) during summer and winter in the past 12 months. Cold water or drinks are defined as water or drinks at a temperature of ≤4 °C, or those consumed immediately after being taken out from the refrigerator, or those that are iced. Hot water or drinks are defined as water of drinks at a temperature of ≥40 °C. They had the option to reply by selecting one of the following provided multiple-choice responses: Never or less than 1 time/month, 1–3 times/month, 1 time/week, 2–4 times/week, 5–6 times/week, 1–2 times/day, 3–5 times/day, or 6+ times/day.

#### 2.2.2. Assessment of Room Temperatures during Winter and Summer

We inquired about the average indoor room temperatures at home and in public places where participants spend most of their time (e.g., school and work). We asked for home and public temperatures during both the winter and summer seasons, as well as during the following three periods in participant lives: the past 12 months, more than 12 months ago but after the age of 18, and before the age of 18. Participants provided their responses using the following temperature ranges: below 65 °F or 18 °C, 65–69 °F or 18–20 °C, 70–74 °F or 21–23 °C, 75–79 °F or 24–26 °C, 80–85 °F or 27–29 °C, and above 85 °F or 29 °C.

#### 2.2.3. Clothing Habits during Winter or Cold Months

We inquired whether participants frequently wear more clothing because they are more sensitive to cold. We also asked whether participants frequently wear thermal pants or leggings under their regular pants during winter and colder months, as many Asian cultures emphasize wearing thermal pants in addition to wearing more clothes. This is important for women, especially during their menstrual periods. Participants indicated their responses to these questions for the preceding 12-month period, using the options of never, rarely, sometimes, often, or always.

### 2.3. Assessment of Cold Hands and Cold Feet

In order to evaluate participants’ sensation of cold hands and feet, we inquired about the frequency at which they encountered this condition in the past 12 months at indoor room temperature. Their responses were categorized into the following options: never, rarely, sometimes, often, or always.

### 2.4. Assessment of Covariates

Demographic characteristics and health status, including chronic conditions were self-reported in our current survey and were collected among new participants. Some of these characteristics encompass age, height, weight, place of birth, and years of living in the U.S. Other health conditions such as insomnia, depression, hypertension, diabetes, cardiovascular diseases, and cancer were collected. Physical activity levels were assessed using a validated questionnaire [24] from the Multiethnic Cohort Study [25]. Assessment of smoking status and intensity were measured from a questionnaire used in the Women’s Healthy Eating and Living Study and has been found to be associated with risk of mortality [26].

### 2.5. Assessment of Dysmenorrhea

We inquired whether women had ever experienced dysmenorrhea in the past. For postmenopausal women, to reduce recall bias, we asked about dysmenorrhea status within the last 5 years of their premenopausal period. If yes, we asked them to rate their level of pain on a scale from 1 to 10, with the following details: 0—No pain, no discomfort; 2—Mild pain, hurts a little bit; 4—Moderate pain, hurts a bit more; 6—Moderate pain, hurts even more and often; 8—Severe pain, hurts a lot; 10—Extreme pain, always hurts.

### 2.6. Statistical Analyses

To assess the differences in baseline characteristics, we employed the χ^2^ test for categorical variables and t-test and analysis of variance (ANOVA) for normally distributed continuous variables. We used linear regression models and controlled for confounding factors such as age, body mass index (BMI), physical activity, use of pain relievers, smoking status, smoking intensity, and clothing habits (e.g., wearing thermal pants or additional layers of clothing). We examined associations between cold exposures and dysmenorrhea in Asians, Whites, and other racial groups, separately. We further stratified the analysis by their residential states (i.e., California, other states) or by migrant status (i.e., Asian migrants and Asian non-migrants). Dysmenorrhea score was treated as a continuous variable as the residual distribution was normal [27]. To evaluate the statistical significance of different strata, such as whether there is any interaction between race, geographic location, or migrant status in relation to our exposure of interest, we used the Wald *p*-value for the interaction term in a model that also included main effects to assess these interactions.

## 3. Results

### 3.1. Baseline Characteristics of Women in the Dysmenorrhea Study of the HAS

As shown in Table 1, Asian women, enrolled in this study were younger than White and other ethnic groups (mainly Hispanics and Blacks). Most of Asian women were premenopausal women with lower BMI and physical activity levels. Compared to Whites and other racial groups, Asian women had fewer past and current smokers and contraceptive users. Across all ethnic groups, at least 40% of the participants were from California, while 18–19% were from southern states, and 8–13% were from midwestern states. Among those enrolled from California, most of them were residents of Southern California. The distribution across different states was different among various ethnic groups. The *p*-values were <0.01 or 0.001 for all the above comparisons.

### 3.2. Other Baseline Characteristics

Table 2 illustrates the distribution of dysmenorrhea, pain reliever users, and different types of cold exposures across Asians and other racial groups. The survey is a cross-sectional survey, so most cold exposures presented in Table 2 reflect exposures in the past 12 months. As for postmenopausal women, we asked about their consumption of cold drinks or water during the menstrual period within the last 5 years of their premenopausal period.

As shown in Table 2, Asian women had lower percentages of moderate levels of dysmenorrhea and lower use of pain relievers such as anti-inflammatory drugs. The frequency of not or rarely drinking cold water or drinks during their menstrual period was higher in Asians (57%) than other racial groups (39% for Whites and 35% for other races). Whites (66%) and other racial groups (66%) had a higher prevalence of habitually drinking cold water or other drinks, including fruit juice, beverages, milk, and tea, during winter, compared to Asians (49%). *p*-values for the above comparisons were below 0.05. Furthermore, other racial groups appeared to have a higher percentage of women who ate ice cream more than 3 times/week than Whites and Asians (*p* = 0.06).

Though the following cold exposures and dressing habits were not statistically different across different racial groups, there were some differences of the distribution on the following exposures. White women had higher percentages of individuals living in homes with temperatures lower than 70 °C and reported wearing more clothes compared to Asians and other racial groups. However, Asians and other racial groups had a higher percentage of women wearing thermal pants compared to White women. In addition, we also asked similar types of exposures during the summer, but the results are not shown.

### 3.3. The Associations of Cold Exposures in Relation to Dysmenorrhea in White and Asian and across Different Geographic Locations (Table 3)

The dysmenorrhea score was utilized, allowing the beta estimates in the tables to reflect the associations between cold exposures and the severity of dysmenorrhea. We conducted stratified analyses among White and Asian as *p*-value for races for all the three exposures listed in Table 3 were either significant or marginally significant (*p* ≤ 0.1). Within White or Asian strata, *p* values for interaction between states were at least marginally significant for the exposures that were significant associated with dysmenorrhea (*p* ≤ 0.1).

**Table 3:** Cold exposures in relation to dysmenorrhea overall and by different geographical locations.

			White			Asian			
	*p* for Race	Overall	California	Other States *	*p* for State	Overall	California	Other States	*p* for State
	Interaction	Beta (*p*-Value)	Beta (*p*-Value)	Beta (*p*-Value)	Interaction	Beta (*p*-Value)	Beta (*p*-Value)	Beta (*p*-Value)	Interaction
Drink cold water or drinks in winter **									
(Cold water >5 times/d or cold drink >4 times/d)									
No		Ref	ref	Ref		Ref	Ref	Ref	
Yes	**0.07**	**0.48 (0.05)**	−0.001(1.2)	**0.96 (0.03)**	**0.03**	0.14(0.40)	0.37 (0.17)	0.04 (0.9)	0.85
**Frequency of ice cream consumption in winter**									
Never		Ref	Ref	Ref		Ref	Ref	Ref	
1-3 times/month		0.12 (0.60)	0.10 (0.70)	0.33 (0.51)		0.10 (0.60)	−0.23 (0.39)	0.39 (0.28)	
1 time/week		−0.08 (0.74)	0.06 (0.92)	−0.18(0.64)		0.39 (0.20)	0.46 (0.27)	0.40 (0.40)	
more than 3 times/week	**0.03**	−0.40 (0.36)	−0.70 (0.18)	−0.65 (0.44)	0.33	**1.23 (0.001)**	**1.34 (0.07)**	**2.5 (0.01)**	**0.07**
**Home room temperature in winter in the past 12 months**									
<65 °F or 18 °C		Ref	Ref	Ref		Ref	Ref	Ref	
65–69 °F or 18–20 °C		**−0.86 (0.05)**	−0.81 (0.14)	**−0.64 (0.02)**		0.41 (0.24)	0.34 (0.39)	0.88 (0.26)	
70–74 °F or 21–23 °C		**−0.94 (0.02)**	**−0.50 (0.04)**	**−1.67 (0.02)**		**0.55 (0.10)**	0.06 (0.88)	1.05 (0.16)	
≥70–74 °F or 21–23 °C	**0.06**	**−1.00 (0.05)**	**−0.45 (0.08)**	**−1.56 (0.04)**	**0.09**	**0.66 (0.08)**	0.40 (0.55)	1.10 (0.21)	0.87

* Other states include southern states (e.g., Alabama, Arkansas, Washington D.C., Kentucky, Louisiana, Maryland), Midwestern states (e.g., Illinois, Indiana, Kansas, Missouri, Nebraska, North Dakota, Ohio), Northeastern states (e.g., Massachusetts, New Hampshire, New York, Pennsylvania) ** ‘Cold water or drinks’ refers to the water or beverages that are close to 4 °C (iced) or that have just been taken out of the refrigerators before consumption. Covariates include age, body mass index, physical activity smoking status and pack-years for past and never smokers, use of oral contraceptives, and use of anti-inflammatory pain reliever. Bolded results are usually marginally (*p* ≤ 0.10) and statistically significant (*p* < 0.05).

#### 3.3.1. Cold Water/Drinks during Winter

We present results for Whites and Asians because most of the associations were not significant for other racial groups likely due to small sample size. Overall, as shown in Table 3, we observed a significant and positive association between the consumption of cold water/drinks during winter and dysmenorrhea among White women in both the entire dataset and in other states but not in California. Here, “other states” refers to states with four distinct seasons, namely the South, Midwest, and Northeast regions. The specific southern states in this study did not include states such as Florida, which may not have distinct seasons (see footnotes for Table 3 for a list of specific southern states). Compared to the ‘No’ category, the beta estimates for women in the ‘Yes’ category (those drinking cold water more than 5 times a day and cold drinks more than 4 times a day) were 0.48 (*p* = 0.05) for the whole dataset and 0.96 (*p* = 0.03) for the other states. We did not observe significant associations between cold water/drinks and dysmenorrhea among Asians.

#### 3.3.2. Ice Cream Consumption during Winter

We found a higher frequency of ice cream consumption in winter to be significantly associated with a greater degree of dysmenorrhea in Asian women. These associations were significant across the entire dataset, in other states, and in California, with the strongest associations observed in the other states. Compared to women who never consumed ice cream in winter, those who ate ice cream more than 3 times a week during the winter season had a beta estimate of 1.23 (*p* = 0.001) in the entire dataset; the beta estimate was 1.34 (*p* = 0.07) for those living in California and 2.5 (*p* = 0.01) for those in other states. No significant associations were found between ice cream consumption and dysmenorrhea among White women.

#### 3.3.3. Home Room Temperature during Winter in the Past 12 Months

In the past 12 months, an increased home room temperature during winter was associated with reduced severity of dysmenorrhea in White women. Among White women, when comparing those who lived in home room temperatures below 65 °F (18 °C) to those who lived in temperatures higher than 70–74 °F (20–23 °C), the latter group had lower estimates: beta = −1.00 and *p* = 0.05 for the entire data set (across all states), beta = −0.45 and *p* = 0.08 for those living in California, and beta = −1.56 and *p* = 0.04 for those living in other states.

We observed an opposite trend among Asian women. An increase in home room temperature during winter over the past 12 months was associated with a heightened severity of dysmenorrhea among Asian women. The beta estimate was 0.66 and *p* = 0.08 overall (across all states) when comparing women who livered in temperatures higher than 70 °F–74 °F (20–23 °C) than those who lived in home room temperatures below 65 °F (18 °C). Although no significant associations were found for those living in California or other states, the trends were consistent with overall results in Asians.

We also examined the room temperature during the periods before 12 months with dysmenorrhea and found that the trends were similar as the trend for recent past 12 month (results not shown). We also examined the room temperatures in public places during winter; however, the results were not significant.

Finally, for the associations examined above, associations with dysmenorrhea were observed only with cold exposures in winter but not in summer (data not shown).

### 3.4. The Associations between Cold Exposures and Dysmenorrhea among Women with Different Status of Cold Hands and Cold Feet

As shown in Table 4, we found that Asian women who sometimes, often, or always had cold hands and feet exhibited increased severity of dysmenorrhea if they consumed ice cream more frequently during winter (beta = 0.21; *p* = 0.03). Similarly, among White women who reported having cold hands and feet (sometimes, often, or always) and had a higher frequency of consuming cold water/drinks in winter, there was an elevated severity of dysmenorrhea (beta = 0.72; *p* = 0.01) compared to those who did not frequently consume cold water/drinks. The above associations were not significant for individuals who never or rarely experienced cold hands or feet. We did not observe a significant disparity in relation to cold hands and feet status when examining room temperature in winter among Whites, even though we did observe a significant association between room temperature and dysmenorrhea overall and in some geographic locations among Whites. *p*-values for interaction between the two cold-hand–cold-feet strata were significant for ice cream and cold drinks/water exposures (*p* < 0.05).

We have presented the results only for racial groups that exhibited significant discrepancies by status of cold hands and feet in Table 4. For instance, as no significant difference was found for ice cream consumption among White women, these results were not presented.

### 3.5. Significant Associations between Several Cold Exposures and Dysmenorrhea Contrary to Our Hypotheses and Racial Disparities

We identified several significant associations that were contrary to our hypotheses (Table 5). However, the types of cold exposures significantly associated with dysmenorrhea vary between Whites and Asians. Among Asians, we found that women who always drank cold water during their period were less likely to experience severe dysmenorrhea (beta = −0.63, *p* = 0.05) compared to those who never drank cold water during that time. Additionally, Asian women who always wore thermal pants on cold days were more likely to experience severe dysmenorrhea (beta = 0.99, *p* = 0.01) than those who never did.

For White women, although we did not find significant associations with the aforementioned exposures, we did discover that those who always wore more clothes than others were more likely to experience severe dysmenorrhea (beta = 1.09, *p* = 0.02) compared to those who never did. Among all the three exposures, *p* for interaction was only marginally significant for wearing thermal pants between Asians and Whites.

### 3.6. Disparities between Cold Exposures and Dysmenorrhea between Asian Migrants and Asian Non-Migrants (Table 6)

We found that Asian migrants who always drank cold water during their menstrual period were less likely to experience severe dysmenorrhea (beta = −1.9 and *p* = 0.005) compared to Asian migrants who never did. However, we did not observe this pattern among Asian non-migrants. *p*-value for interaction between the two strata = 0.02. Asian migrants who always wore thermal pants during cold days were more likely to experience severe dysmenorrhea (beta = 1.76 and *p* = 0.0009) than those who never did. Asian non-migrants appear to exhibit similar trends, albeit with lower magnitudes and less significance. *p*-value for interaction between migrant and non-migrant was not significant.

**Table 6:** Impacts of acculturation among Asian immigrants—disparities in cold exposures and dysmenorrhea between Asian migrants and Asian non-migrants.

	Overall Beta (*p*-Value)	Migrant Beta (*p*-Value)	Non-Migrant Beta (*p*-Value)	*p*-Value of Interaction
Cold water/drinks * during menstrual period				
Never	Ref	Ref	Ref	
Rarely	−0.29 (0.23)	**−0.64 (0.10)**	−0.31 (0.30)	
Sometimes	**−0.41 (0.10)**	**−0.96 (0.04)**	−0.20 (0.51)	
Often	**−0.50 (0.08)**	**−0.28 (0.05)**	−0.40 (0.26)	
Always	**−0.63 (0.05)**	**−1.9 (0.005)**	0.26 (0.51)	**0.02**
**Wearing thermal pants during cold months**				
Never	Ref	Ref	Ref	
Rarely	**0.39 (0.09)**	**1.05 (0.02)**	0.10 (0.71)	
Sometimes	0.12 (0.67)	0.02 (0.95)	0.34 (0.32)	
Often	**0.98 (0.001)**	0.87 (0.14)	0.42 (0.32)	
Always	**0.99 (0.01)**	**1.76 (0.0009)**	**1.00 (0.06)**	0.22

* ‘Cold water or drinks’ refers to the water or beverages that are close to 4 °C (iced) or that have just been taken out of the refrigerators before consumption. Covariates include age, body mass index, physical activity, smoking status, and pack-years for past and never smokers, use of contraceptives, and use of anti-inflammatory pain reliever. Bolded results are usually marginally (*p* ≤ 0.10) and statistically significant (*p* < 0.05).

## 4. Discussion

Overall, our study is the first to provide significant evidence supporting our hypotheses. These findings indicate that greater cold exposures are positively associated with more severe dysmenorrhea, particularly during winter. For instance, a higher frequency of consuming cold water/drinks and ice cream in winter, as well as having a colder room temperature at home in winter, was associated with the severity of dysmenorrhea. These associations were stronger among women living in regions with distinct seasons but were weaker in California.

On the other hand, several significant associations between cold exposures and dysmenorrhea contradicted our hypotheses. These contradictions could arise from reverse causation, cultural influences, or individual susceptibilities, which will be discussed later.

We first would like to discuss potential biological mechanisms that may support our hypotheses. Cold exposure constricts blood vessels, reducing blood flow and oxygen supply to the affected area, resulting in tissue damage, inflammation, and pain [28,29,30,31]. Though not thoroughly studied in humans, animal studies have shown that cold stress extends the estrous cycle, causing reproductive hormone disorder and microcirculation disturbance [13]. Each of these factors has been linked with dysmenorrhea in humans [10,32]. Cold exposures also stimulate the release of prostaglandins, reduce perfusion of microcirculation [11], lower nitric oxide levels, which can reduce blood flow in the uterus [12]. All of these factors can increase sensation of pain [6,33]. Additionally, prostaglandins make blood vessels leaky, amplifying the sensation of cold and further contributing to the perception of pain [34].

In addition to external cold-temperature exposures, some studies on the effects of cold/warm drinks on other outcomes can provide insights into potential mechanisms related to dysmenorrhea. For instance, research conducted on early post-weaning rabbits during winter has demonstrated that rabbits consuming warm water in winter experienced increased growth, reduced diarrhea, lowered jejunal mRNA expression of pro-inflammatory cytokines (TGF1, IL-1β, and IL-12), and an increased relative abundance of beneficial cecal microorganisms like *Coprococcus* spp., as compared to rabbits consuming cold water in winter [35]. Inflammation, as introduced earlier, is associated with dysmenorrhea [28,29,30,31]. While gut microbes do not inhabit the uterus, studies have indicated a connection between gut microbes and endometriosis, a potential downstream outcome of dysmenorrhea [36,37]. Disrupted gut microbes can secrete metabolites that influence the immune and circulatory systems, potentially altering the environment to favor inflammation and promoting endometriosis, a condition that can cause dysmenorrhea symptoms [36,37].

The influence of cold exposures on dysmenorrhea depends on season and geographic location. The above papers also focus on the winter season [35]. In winter, warm-blooded mammals, like humans, need to perform thermogenesis through increased metabolic rate and peripheral blood vessel constriction to maintain core body temperature [28,38]. Hence, additional cold exposure in winter can further burden thermogenesis and reduce blood circulation [28]. For instance, in a cold environment, prolonged consumption of cold water/beverages in winter with wearing fewer clothes in winter will further deplete stored energy for thermogenesis, leading to reduced blood flow in the uterus [13] and in the extremities resulting in cold hands and feet [10,28]. From a natural physics point of view, blood vessels become more constricted and stiffer in cold temperatures, and blood pressure often increases in winter [39,40,41].

We observed that the effects of cold exposure on dysmenorrhea were more pronounced in states with colder winters compared to California. Our study further reinforces the idea that the influence of cold exposures varies depending on the season, as we did not find any associations of the same exposures during summer. Cold water during summer may not be that harmful but may be beneficial, especially during exercise [42,43].

The impact of cold exposure on dysmenorrhea may hinge on individual susceptibilities. Our study found that women with cold hands and feet are more susceptible to cold-induced dysmenorrhea during winter, confirming TCM self-care philosophy. This suggests that those with compromised peripheral circulation and potentially reduced thermogenesis capacity are more likely to experience dysmenorrhea triggered by cold beverages or ice cream. This evidence aligns with the previously discussed dysregulated microcirculation association with dysmenorrhea [13].

Some significant associations, which are contrary to our hypotheses, likely result from reverse causation and cultural influences. Asian women, influenced by traditional Asian culture and principles of TCM, often avoid cold exposure during menstruation to ease symptoms like severe dysmenorrhea. TCM emphasizes avoiding coldness in the lower body. This leads to behaviors such as wearing thermal pants, which is more common among Asians and Asian migrants compared to Whites and Asian non-migrants. Asian migrants, having been deeply influenced by their upbringing in Asia, tend to follow these cultural norms more closely than non-migrants.

On the other hand, we found that Whites were impacted by a different kind of culture. It appears that White women who experienced more severe dysmenorrhea are more likely to cope with cold exposures by wearing more clothes, rather than opting for thermal pants or avoiding cold water during menstrual periods. These behaviors are consistent with the prevailing cultural practices in the U.S., where cold drinks and water are more commonly consumed than in Asian countries [44].

It is worthwhile to mention the positive association between ice cream consumption in winter and dysmenorrhea among Asians. Although Asian culture advises women not to consume cold drinks in winter, some Asians may consider that consuming ice cream only a few times a week will not cause much harm. The null association among Whites regarding this connection is likely because Whites are less susceptible to cold exposures when the consumption is only a few times a week, rather than daily. However, increased consumption of cold water/drinks a few times a day can eventually lead to negative effects on Whites, as seen in this study.

While we discovered an association between cold water/drinks and water/beverages, we did not find a similar association related to cold foods. One possible explanation could be that the frequency of water and drink consumption is generally higher than that of foods, particularly cold foods. More than 70% of women reported consuming cold meals once a week or less, which could account for the absence of the observed association.

Our study holds several strengths: it is the first investigation of cold exposures in relation to dysmenorrhea among the general population including white and Asian women, extending beyond just occupational women in slaughter houses [5]. Comparing the strengths of the associations across states with more extreme climates to California further strengthens our hypothesis. Furthermore, our studies have established connections between epidemiologic results and evidence derived from animal models and human physiology studies [13,28]. This approach will provide valuable evidence for subsequent mechanistic studies. Moreover, Asians constitute the largest segment of the global population. Certain Asian cultural practices have been indirectly linked to reduced risks of breast and colon cancer, as evidenced by the contrast in risk levels between Asian immigrants and their native counterparts in the case of these cancers [45]. Thus, an exploration of Asian cultural practices in tandem with health outcomes can not only elucidate disease mechanisms and racial disparities between Asians and non-Asians but also potentially leverage these practices to prevent dysmenorrhea in other ethnic groups.

Our study certainly has several limitations. As a cross-sectional study, a causal relationship cannot be established. However, it provides valuable evidence to design prospective studies for further validation of our hypothesis. Cross-sectional studies can be strongly influenced by reverse causation, leading to associations that contradict our hypotheses. Our study is also subject to recall bias, particularly in the case of dysmenorrhea among postmenopausal women, who may not recall their past status as accurately as premenopausal women. Because the age distribution differs between Asians and Whites, differential recall bias cannot be avoided between the two groups. Future studies focusing on premenopausal women with a similar age range across ethnic groups will ensure more efficient comparisons. Residual confounding and measurement errors also existed. For instance, our not observing an association between room temperatures at public places is likely due to measurement error. Women are more likely to recall the temperatures at home rather than in public places where the temperatures were centrally controlled. Dr. Tianying Wu designed the questionnaires related to cold exposure. Although demonstrating associations between these cold exposures and outcomes is one way to validate these questions, future studies with objective measures can further strengthen the validity. We have used different strategies to sample our participants, such as through various organizations, alumni and friend networks, national registry data, etc. These sampling methods are not entirely random and could result in selection bias. Due to smaller sample size, some *p*-values for interaction were not significant even we found significant trends within certain strata. Large-scale studies are needed to repeat our findings. Nevertheless, these findings contribute to the generation of new research questions that will enable a deeper exploration of reverse causation and the impacts of acculturation.

## 5. Conclusions

In summary, dysmenorrhea is significantly understudied but is highly prevalent among women. It not only causes severe pain and leads to work and study absences, but it also serves as an early sign for many disorders in the reproductive system, such as endometriosis, infertility, and pelvic inflammatory disease. If cold exposures, like consumption of cold water and ice cream in winter can lead to dysmenorrhea, this problem can be easily addressed. Identifying non-pharmacological approaches to address dysmenorrhea will offer both cost-effective solutions and wider applications for women and teenage girls. Identifying disparities between Asians and non-Asians also aids in recognizing risk factors associated with race, thus facilitating the development of racially specific prevention strategies.

## Figures and Tables

**Table 1:** General Characteristics of Women in the Dysmenorrhea Study from the Healthy Aging Study.

	White (*n* = 148)	Asian (*n* = 197)	Other Races (*n* = 74)	*p*-Value
**Age** (Median, inter-quartile range)	49 (31–59)	30 (25–40)	32 (26–45)	<0.0001
**BMI** (Median, inter-quartile range)	23.7 (20.9–29.0)	22.5 (20.5–26.0)	24.8 (22.0–28.5)	0.0008
**Physical activity** (MET-hour/week) *	6.4 (3.5–9.7)	4.4 (2.4–8.0)	5.8 (3.4–9.6)	0.002
(Median, inter-quartile range)				
**Migrant** (N, %)				
No	144 (97)	127 (64)	71 (96)	
Yes	4 (3)	70 (36)	3 (4)	<0.0001
**Smoking status** (N, %)				
Never smoker	111 (75)	185 (94)	57 (77)	
Past smoker	27 (18)	11 (5.5)	10 (14)	
Current smoker	10 (7)	1 (0.5)	7 (9)	<0.0001
**Menopausal status** (N, %)				
Premenopausal	65 (44)	168 (85)	52 (70)	
Perimenopausal	9 (6)	17 (9)	5 (7)	
Postmenopausal	74 (50)	12 (6)	17 (23)	<0.0001
**Use of oral contraceptive** (N, %)				
Yes	126 (85)	118 (60)	53 (72)	<0.0001
No	22 (15)	79 (40)	21 (28)	
**U.S. states** (N, %)				
California	69 (47)	79 (42)	46 (62)	
Southern	26 (18)	36 (19)	13 (18)	
Midwestern	14 (9)	24 (13)	6 (8)	
Northeastern	29 (13)	42 (22)	6 (8)	0.005

* MET-hour for moderate and vigorous physical activity/week. Continuous variables are presented as median; (inter-quartile range). Categorical variables are presented as N (%). Abbreviations: BMI = body mass index; METs = metabolic equivalents.

**Table 2:** Dysmenorrhea, uses of pain medications, and types of cold exposures across different racial groups.

	White (*n* = 148)	Asian (*n* = 197)	Other Races (*n* = 74)	*p*-Value
**Dysmenorrhea level** (N, %)				
No pain (score 0 to <2)	19 (12)	38 (19)	19 (26)	
Mild pain (score 2 to <4)	31 (20)	62 (32)	19 (26)	
Moderate pain (score 4 to <8)	78 (50)	79 (40)	28 (37)	
Severe pain score (score 8 to 10)	20 (13)	18 (13)	8 (11)	**0.03**
**Medication** (N, %)				
Acetaminophen (e.g., Tylenol, Panadol)	97 (66)	118 (60)	52 (70)	0.24
Anti-inflammatory pain reliever (e.g., Ibuprofen, Advil, Motrin)	121 (82)	136 (69)	58 (78)	**0.02**
**Frequency of drinking cold water/drinks during menstrual period**				
Never or rarely	57 (39)	112 (57)	26 (35)	
Sometimes	52 (35)	42 (21)	30 (40)	
Often or always	39 (26)	43 (22)	18 (25)	**0.004**
Habits of drinking ‘cold water or drinks’ * in winter				
**(Cold water > 5 times/d or cold drinks > 4 times/d in winter)**				
No	50 (34)	100 (51)	25 (34)	
Yes	98 (66)	97 (49)	49 (66)	**0.002**
**Frequency of ice cream consumption in winter**				
Never	75 (51)	75 (38)	27 (36)	
1–3 times/month	39 (26)	74 (38)	23 (31)	
1 time/week	18 (12)	27 (14)	12 (16)	
More than 3 times/week	16 (11)	21 (10)	12 (17)	**0.06**
**Home room temperature < 70 °F in winter**				
Yes	77 (52)	90 (46)	35 (47)	
No	71 (48)	107 (54)	39 (53)	0.5
**Wear more clothes than others**				
No or rarely	57 (38)	81 (40)	35 (48)	
Sometimes	50 (34)	70 (36)	23 (31)	
Often or always	41 (28)	46 (24)	16 (21)	0.68
**Wear thermal pants during cold months**				
No or rarely	103 (68)	132 (67)	45 (61)	
Sometimes	24 (16)	31 (16)	14 (19)	
Often or always	21 (14)	34 (17)	15 (20)	0.52

Data in each column are presented in N (%). * ‘Cold water or drinks’ refers to the water or beverages that are close to 4 °C (iced) or have just been taken out of refrigerators before consumption. Bolded *p*-values are usually marginally (*p* ≤ 0.10) and statistically significant (*p* < 0.05).

**Table 4:** Cold exposures in relation to dysmenorrhea by status of cold hands and cold feet.

	Status of Cold Hands and Cold Feet		
	Never or Rarely	Sometimes, Often, or Always	*p*-Value for Interaction
	Beta (*p*-Value)	Beta (*p*-Value)	
Frequency of ice cream consumption * in winter among Asians	0.11 (0.48)	**0.21 (0.03)**	**0.02**
Whites who drank cold water/drinks more frequently vs. those who drank them less often ** during winter	−0.30 (0.43)	**0.72 (0.01)**	**0.008**
Degree of home room temperature * in winter in the past 12 months among Whites	−0.09 (0.58)	−0.16 (0.28)	0.81

* Indicates that the exposure variables were entered as ordinal variables. ** We compared two groups of Whites (those who drink cold water more than 5 times/day or more than 4 times/day vs. those who did not). ‘Cold water or drinks’ refers to the water or beverages that are close to 4 °C (iced) or that have just been taken out of the refrigerators before consumption. Covariates include age, body mass index, physical activity, smoking status, and pack-years for past and never smokers, use of oral contraceptives, and use of anti-inflammatory pain reliever. Bolded results are usually marginally (*p* ≤ 0.10) and statistically significant (*p* < 0.05).

**Table 5:** Significant associations that are contrary to our hypotheses—disparities in types of cold exposures with dysmenorrhea between Whites and Asians.

	White	Asian	*p* for Interaction
	Beta (*p*-Value)	Beta (*p*-Value)	
Drinking cold water/drinks * during menstrual period			
Never	Ref	Ref	
Rarely	0.19 (0.22)	−0.29 (0.23)	
Sometimes	0.11 (0.09)	**−0.41 (0.10)**	
Often	0.33 (0.32)	**−0.50 (0.08)**	
Always	−0.33 (0.30)	**−0.63 (0.05)**	0.21
**Wearing more clothes than others**			
Never	Ref	Ref	
Rarely	0.26 (0.40)	−0.15 (0.6)	
Sometimes	**0.95 (0.004)**	−0.18 (0.51)	
Often	0.28 (0.41)	0.03 (0.93)	
Always	**1.09 (0.02)**	0.35 (0.43)	**0.1**
**Wearing thermal pants during cold months**			
Never	Ref	Ref	
Rarely	0.18 (0.59)	**0.39 (0.09)**	
Sometimes	−0.20 (0.48)	0.12 (0.67)	
Often	0.20 (0.60)	**0.98 (0.001)**	
Always	0.81 (0.12)	**0.99 (0.01)**	**0.06**

* ‘Cold water or drinks’ refers to the water or beverages that are close to 4 °C (iced) or that have just been taken out of the refrigerators before consumption. Covariates include age, body mass index, physical activity, smoking status, and pack-years for past and never smokers, use of contraceptives, and use of anti-inflammatory pain reliever. Bolded results are usually marginally (*p* ≤ 0.10) and statistically significant (*p* < 0.05).

## Data Availability

Our study is still in the pilot state, and data for this paper are not available to share.
